# Annotations

**Published:** 1905-08-26

**Authors:** 


### ANNOTATIONS.

### Post-Graduation Vacation Study.

The development of post-graduation studies is certainly one of the features of modern medical history, and it may be taken for granted that here, as we have recently been told is already the case on the other side of the Atlantic, it will gradually be recognised that such studies have a commercial as well as a scientific value. The schemes already existing in London have undoubted merits, and we are glad to know that they can claim a fair measure of success. It is certain that the work in which they are engaged is of high importance in relation to the public not less than to the profession. But if they are fully to utilise their opportunities it is essential that their plans be adapted to the convenience of the general practitioner who is, indeed, their immediate care. Now it is notorious that many practitioners cannot leave their practices apart from the few weeks which they devote to their annual holiday, and, whether physiologically wise or not, it cannot be doubted that some would be willing to spend these weeks in London, if by so doing they could obtain efficient teaching and opportunities for practical work. In many, probably in the majority of instances, the time which could thus be spent would be in the month of August, when, for the most part, hospital and teaching work in London is reduced to a minimum. Here then it would seem is the opportunity of the post-graduation organisations. In spite of the necessary absence of some prominent names during the holiday month, there are numbers of highly competent and authoritative teachers who might be induced to take part in a scheme which would, we feel sure, attract numerous country practitioners. And if the several organisations would co-operate for such a purpose they would not only be engaged in work in harmony with their proper function, but they might also reap a not unsubstantial reward. The time for such a suggestion may not seem the most appropriate, but if it is to be carried into practice in an efficient manner careful arrangements must be made long in advance, and the deficiency of the present year is a suitable opportunity for considering how the gap may be filled in the future.

### Fees under the Midwives Act.

The Midwives Act of 1902 excited at the time considerable difference of opinion in the medical profession, but as it is now enrolled on the Statute Book, nothing remains but honestly to carry out its provisions. It has, however, long since become evident that there exists one important omission—namely, lack of power on the part of local authorities to pay for services rendered under the direction of the Act. The midwife, when sending for a medical practitioner in one or other of the various emergencies which may arise during the process of labour or in the course of the puerperium, is not taking the step on her own choice or discretion. On the contrary, she is carrying out the instructions of an Act of Parliament which is administered by the local authorities; but for the remuneration of the medical practitioner summoned in these circumstances no provision is made. The London County Council proposes in its next General Purposes Bill to apply to Parliament for powers to remedy this defect. It is important that the terms of the proposed clause shall be carefully scrutinised. Suggestions have been made that such payments should be made only in cases where the practitioner fails to obtain his fee from the patient. But such a position cannot be defended. The medical man is not summoned by the patient but by the midwife, and the midwife acts under the license of the local authority. The medical man in attending is responding, not to a private, but to a public claim, and it is to the public representatives he must look for his fee. If the local authority is able to recover from the patient the sum paid to the practitioner, so much the better in the interests of the ratepayers and of sound economics. But with that the medical practitioner, as such, has no concern. His services are claimed by a representative of the public authorities, and it is to these authorities he must, therefore, look for the payment of his fees.

### Sanatoriums and Friendly Societies.

In the recent discussion at Leicester on the provision of sanatoriums for phthisical patients of the poorer classes, some interesting particulars were announced of a scheme which both on medical and economic grounds has much to commend it. The proposal is the outcome of a visit which was paid some little time since by representatives of the leading English Friendly Societies, in conjunction with Dr. A. P. Hillier, to the German industrial sanatoriums. The plan, as now defined, is to erect a sanatorium capable of accommodating 200 patients, and it is intimated that a site having an extent of 250 acres has already been secured for this purpose by the representatives of friendly societies whose total constituents number some 2,000,000 workers. To the initial expenses friends of the societies are being asked to contribute, but no cost for maintenance will fall on the charitable public. It is an essential feature of the scheme that before the institution is opened every one of the 200 beds will be reserved, endowed, and maintained by a levy on the societies and organisations affiliated for the purposes of the scheme. The Post Office, it is announced, has consented to join the movement. In all this there is much cause for satisfaction, and that, not only in the proposal itself, but in the sane and healthy spirit which has prompted it and the moral certainty that so fair an example will not lack imitation. The sanatorium treatment has probably not yet reached its true therapeutic level, but it is of undoubted value, and it especially needs at present an adequate presentation in its economic aspect. Above all, it is decidedly to the good that a scheme for securing its benefits for the industrial classes is to be organised by capable representatives of these classes, and is to be financed on the sound principle of mutual insurance. Under such an arrangement there need be no hesitation in believing that good business management will be allied with effective medical direction and supervision.

